# The effect of web-based educational intervention on caries-preventive oral health behaviors in pregnant women: an application of the health belief model

**DOI:** 10.1186/s12889-026-26315-6

**Published:** 2026-02-11

**Authors:** Sedigheh Kheirandish, Homamodin Javadzadeh, Marzieh Mahmoodi, Seyed Hossein Mousavi, Hadi Abbasi

**Affiliations:** 1 Department of oral and maxillofacial pathology, School of Dentistry, Bushehr university of medical sciences, Bushehr, Iran; 2 Department of Health Education and Health Promotion, School of health and nutrition, Bushehr University of Medical Sciences, Bushehr, Iran; 3 Addiction and Lifestyle Research Center, Bushehr university of medical sciences, Bushehr, Iran; 4 Department of Biostatistics and Epidemiology, School of health and nutrition, Bushehr university of medical sciences, Bushehr, Iran; 5 Department of Medical Emergencies, School of Allied Medical Sciences, Bushehr University of Medical Sciences, Bushehr, Iran; 6 School of Dentistry, Bushehr university of medical sciences, Bushehr, Iran

**Keywords:** Health belief model, Oral health, Pregnancy, Web-based education, Preventive behaviors

## Abstract

**Background:**

Oral health during pregnancy is critical for both maternal and neonatal outcomes, yet awareness and preventive behaviors remain suboptimal. This study evaluated the effect of a web-based educational intervention, grounded in the Health Belief Model (HBM), on caries-preventive oral health behaviors in pregnant women.

**Methods:**

In a quasi-experimental design, 66 pregnant women in Bushehr, Iran, were randomly assigned to intervention and control groups. The intervention group received a multimedia web-based education program based on HBM constructs, while the control group received routine care. Data on knowledge, HBM constructs, and preventive behaviors were collected before and three months after the intervention using validated questionnaires. Statistical analyses included repeated measures ANOVA to compare changes over time between groups.

**Results:**

Post-intervention, the intervention group showed significant improvements in knowledge and most HBM constructs (perceived susceptibility, severity, benefits, barriers, and self-efficacy) compared to controls (*p* < 0.05). Although preventive oral health behaviors increased significantly within the intervention group (*p* = 0.022), between-group differences in behavior change were not statistically significant (*p* = 0.171).

**Conclusion:**

The web-based educational program effectively enhanced pregnant women’s knowledge and health beliefs regarding oral health but did not produce a statistically significant improvement in preventive behaviors compared to routine care. Integrating HBM-based web education offers a flexible, cost-effective approach to promote oral health awareness during pregnancy, though further strategies may be needed to translate knowledge gains into sustained behavioral change.

## Background

In recent decades, oral health during pregnancy has gained recognition as an essential component of maternal and neonatal healthcare [1]. The World Health Organization (WHO) identifies oral health as a key preventive priority for this population [2]. Physiological and behavioral changes during pregnancy can adversely affect oral health. For instance, increased hormone levels have been linked to declining periodontal health, with approximately 60–75% of pregnant women experiencing gingivitis [1, 3].

The most common oral changes during pregnancy include gestational gingivitis (prevalence: 60–75%) [4], xerostomia (15–18%) [5], pregnancy epulis (5%), dental erosion (75–80%), and halitosis (~ 13%) [6]. These changes not only affect maternal quality of life but are also associated with complications such as preeclampsia, preterm birth, and low birth weight [3, 7]. Placental inflammation reduces the secretion of key growth factors for the fetus, including fibroblast growth factor (FGF) and brain-derived neurotrophic factor (BDNF). Systemic inflammatory responses are also elevated in pregnant women with periodontitis. Moderate to severe periodontitis correlates with higher levels of C-reactive protein (CRP) and prostaglandin E2 (PGE2), which are significant risk factors for adverse pregnancy outcomes [8, 9]. However, awareness of these associations among both women and healthcare providers remains inadequate, leading to neglect of oral health’s critical role in fetal development [10, 11].

Given these findings, preventive programs for vulnerable groups like pregnant women are essential. Studies suggest that oral health education can prevent up to 80% of dental diseases [12]. To achieve this, researchers have employed behavioral change models, such as the Health Belief Model (HBM), which focuses on constructs like perceived threat, perceived benefits/barriers, cues to action, and self-efficacy to motivate preventive behaviors [13].

Information and communication technology (ICT) offers numerous advantages for health management, including improved access to health information, enhanced disease understanding, and reduced complications [14]. Compared to in-person sessions, web-based interventions are flexible, cost-effective, and scalable. Such interventions guided by healthcare professionals (HCPs) via e-counseling or remote monitoring can effectively deliver health education [15].

Although numerous studies have applied the HBM, no domestic research has utilized web-based interventions to improve oral health in pregnant women. This study aimed to design and evaluate an HBM-based web educational program for pregnant women in Bushehr. Its innovation lies in integrating ICT with health education models to enhance accessibility, reduce costs, and increase flexibility.

## Methods

### Study design and participants

This quasi-experimental study employed a pre-test/post-test design with a control group to evaluate the impact of a web-based educational program on preventive behaviors related to dental caries and oral health problems among pregnant women receiving care at comprehensive health service centers in Bushehr, Iran.

Eligibility criteria included: [1] possession of a computer and/or smartphone with internet access [2], being in the first trimester of pregnancy [3], residence in Bushehr, and [4] basic literacy (reading and writing). Exclusion criteria were: [1] employment in a dental-related field [2], having progressive oral or dental diseases [3], unwillingness to participate, and [4] inability to continue in the study due to conditions such as miscarriage.

### Sampling

The sample size was calculated using the formula for comparing the means of two independent groups: $$\:\frac{{({z}_{1-\raisebox{1ex}{$\alpha\:$}\!\left/\:\!\raisebox{-1ex}{$2$}\right.}+{z}_{1-\beta\:})}^{2}({\sigma\:}_{1}^{2}+{\sigma\:}_{2}^{2})}{{({\mu\:}_{1}-{\mu\:}_{2})}^{2}}$$. With a two-tailed type I error (α) of 0.05 and a power (1-β) of 90%, and based on the post-intervention behavioral scores from Nikbin et al. [16] (Control: 49.37 ± 14.6; Intervention: 77.26 ± 9.33), a minimum sample size of 30 participants per group was calculated. Accounting for a potential 10% dropout rate, a total sample of 66 participants (33 per group) was targeted for recruitment.

A two-stage random sampling method was used. First, four out of ten comprehensive health service centers in Bushehr were randomly selected. These four centers were then randomly assigned to either the intervention or control group, resulting in two centers being allocated to the intervention group and two to the control group. Subsequently, from the list of eligible pregnant women in each center, the required number of participants was randomly selected. The number of participants recruited from each center was proportionate to the size of its pregnant population.

Participant lists were extracted from the Integrated Health System (SIB) database, and electronic health records were screened for primary eligibility. From the list of eligible women in each center, the required number of participants was randomly selected and contacted to verify secondary eligibility criteria (internet access, device availability) and willingness to participate. Women who met all criteria and agreed to participate were enrolled. If a woman declined or was ineligible, another participant was randomly selected from the remaining list. This process continued until the target sample size was achieved.

#### Data collection tools

At baseline, after explaining the study objectives and obtaining informed consent, participants completed the study questionnaire, which was adapted from Shamsi et al. [17]. The questionnaire included four sections:

1. Demographic and personal characteristics (11 items).
2. Knowledge (15 true/false items; one point for correct answers, zero for incorrect).
3. HBM constructs (55 items): perceived susceptibility (8 items), perceived severity (7 items), perceived benefits (10 items), perceived barriers (14 items), self-efficacy (8 items), and cues to action (8 items), each scored on a standard 5-point Likert scale.
4. Oral health behaviors (15 items; one point for correct behaviors, zero for incorrect).

Content validity was confirmed by a panel of experts. Internal consistency was acceptable, with a Cronbach’s alpha of 0.84 for the total scale. Subscale alphas were as follows: perceived susceptibility (0.73), perceived severity (0.70), perceived benefits (0.75), perceived barriers (0.70), cues to action (0.73), self-efficacy (0.76), and oral health behaviors (0.88) [17].

### Intervention

The educational content was designed based on the six key constructs of the HBM.

- Perceived susceptibility: included information on the increased risk of dental caries and periodontal disease during pregnancy and their potential effects on maternal and neonatal health.
- Perceived severity: described the possible complications of poor oral hygiene, such as preterm birth, low birth weight, and systemic inflammation, supported by real-life examples and clinical images.
- Perceived benefits: highlighted the advantages of maintaining oral hygiene practices, including regular brushing, flossing, and dental visits, emphasizing their role in preventing complications.
- Perceived barriers: discussed common challenges (e.g., nausea, time limitations, misconceptions about dental care during pregnancy) and practical strategies to overcome them.
- Cues to action: incorporated motivational messages, reminders, and visual prompts throughout the web modules to encourage engagement.
- Self-efficacy: included step-by-step demonstrations, videos, and practical examples to strengthen participants’ confidence in performing oral health behaviors.

The educational package consisted of text, images, short videos, and interactive elements designed to make learning more engaging and accessible.

For the intervention group, a multimedia educational package was uploaded to a dedicated website. Participants were asked to complete the training within one week. Three days after the start, those who had not accessed the website were contacted and encouraged to use the content. At the end of the week, noncompleters were given an additional three days. Participants who did not access the materials within this extended period were excluded from the study.

The control group did not receive the web-based educational content but continued to receive routine education provided by the health centers.

### Follow-up and ethical considerations

Three months after completion of the intervention, participants were reassessed using the same instruments.

After the final assessment, participants in the control group were provided with a username and password to access the educational website, along with a user guide brochure, to ensure equitable access to the intervention content.

To minimize potential measurement bias, both the data collectors and the data analyst were blinded to the group allocation and were unaware of which centers were assigned to the intervention or control groups. All questionnaires were administered by the same trained researcher using standardized instructions. Participants were assured that their responses would remain anonymous and confidential to reduce potential response bias.

### Data analysis

Data were analyzed using SPSS version 24. Descriptive statistics were calculated. Between-group comparisons for categorical variables were conducted using chi-square tests, while independent t-tests were used for continuous variables. Changes in mean scores of the HBM constructs over time between the two groups were examined using repeated measures ANOVA. A p-value of < 0.05 was considered statistically significant.

## Results

A total of 66 pregnant women (33 in each group) were initially enrolled. During the study, one participant from the control group and one from the intervention group withdrew, leaving 64 participants who completed the post-test and were included in the final analysis.

The results showed that the two groups were homogeneous at baseline, with no statistically significant differences in demographic characteristics (P-value > 0.05). Demographic variables included maternal age, spouse’s age, gestational age, DMFT index, mother’s occupation, father’s occupation, insurance coverage, mother’s education, father’s education, pregnancy history, and economic status. Details are presented in Table 1.

**Table 1:** Baseline characteristics of the intervention and control groups

Variable	Control Group	Intervention Group	*P*-value
Mother’s occupation			0.474
Unemployed	26 (81.3%)	29 (90.6%)	
Employed	6 (18.8%)	3 (9.4%)	
Father’s occupation			0.558
Unemployed	0 (0%)	1 (3.1%)	
Employee	11 (34.4%)	10 (31.3%)	
Self-employed	21 (65.6%)	20 (62.5%)	
Retired	0 (0%)	1 (3.1%)	
Insurance coverage			1.000
Yes	29 (90.6%)	29 (90.6%)	
No	3 (9.4%)	3 (9.4%)	
Mother’s education			0.511
Diploma	15 (46.9%)	15 (46.9%)	
Bachelor’s	13 (40.6%)	16 (50%)	
Master’s	3 (9.4%)	1 (3.1%)	
Higher	1 (3.1%)	0 (0%)	
Father’s education			0.601
Diploma	13 (40.6%)	17 (53.1%)	
Bachelor’s	15 (46.9%)	12 (37.5%)	
Master’s	3 (9.4%)	3 (9.4%)	
Higher	1 (3.1%)	0 (0%)	
Pregnancy history			0.599
Yes	22 (68.8%)	20 (62.5%)	
No	10 (31.3%)	12 (37.5%)	
Economic status			0.140
Poor	3 (9.4%)	6 (18.8%)	
Moderate	18 (56.3%)	21 (65.6%)	
Good	11 (34.4%)	5 (15.6%)	
Age (years)	30.63 ± 4.891	29.03 ± 6.281	0.262
Spouse’s age (years)	33.81 ± 4.060	33.09 ± 5.653	0.561
Gestational age (weeks)	13.91 ± 2.401	14.69 ± 1.975	0.160
DMFT index	5.03 ± 4.154	5.31 ± 4.012	0.784

Data are presented as frequency (percentage) and mean ± standard deviation

Before the educational intervention, there were no statistically significant differences between the intervention and control groups in the mean scores of knowledge or any of the Health Belief Model (HBM) constructs (perceived susceptibility, perceived severity, perceived benefits, perceived barriers, cues to action, and perceived self-efficacy) (*P*-value > 0.05).

In the intervention group, significant increases were observed post-intervention in the mean scores for knowledge (*P* < 0.001), perceived susceptibility (*P* < 0.001), perceived severity (*P* = 0.001), perceived benefits (*P* < 0.001), perceived barriers (*P* = 0.007), cues to action (*P* = 0.015), and perceived self-efficacy (*P* < 0.001), compared with pre-intervention scores.

Repeated measures ANOVA comparing changes between the two groups showed that, overall, changes in knowledge and most HBM constructs were significantly greater in the intervention group compared with the control group, except for cues to action, for which no significant difference was found between groups (*P* = 0.290). Similarly, before the intervention, the mean score for preventive oral health behaviors did not differ significantly between the two groups (*P*-value > 0.05). Repeated measures analysis with group and time effects indicated that overall changes in preventive behavior scores over the study period did not differ significantly between the two groups (*P* = 0.171) (Table 2).

**Table 2:** Comparison of knowledge, health belief model constructs, and preventive oral health behaviors before and after intervention in the study groups

Structures	Before	After	*P*-value*	*P*-value**
Knowledge				
Control	7.03 ± 1.78	7.53 ± 2.09	0.136	0.001
Intervention	7.84 ± 1.68	10.72 ± 2.43	< 0.001	
Perceived Sensitivity				
Control	30.96 ± 4.72	32.00 ± 4.63	0.051	0.004
Intervention	31.59 ± 3.18	35.21 ± 4.12	< 0.001	
Perceived Intensity				
Control	28.71 ± 3.08	28.90 ± 2.75	0.612	0.006
Intervention	29.15 ± 2.70	31.37 ± 3.79	0.001	
Perceived Benefits				
Control	42.87 ± 5.98	42.40 ± 5.33	0.386	< 0.001
Intervention	41.65 ± 4.52	45.59 ± 4.37	< 0.001	
Perceived Barriers				
Control	51.43 ± 8.58	49.46 ± 8.10	0.020	0.001
Intervention	50.03 ± 9.17	54.59 ± 9.42	0.007	
Cues to Action				
Control	28.78 ± 5.10	29.68 ± 4.43	0.325	0.290
Intervention	26.38 ± 5.65	28.63 ± 4.76	0.015	
Perceived Self-efficacy				
Control	32.12 ± 3.58	31.06 ± 4.11	0.040	< 0.001
Intervention	31.21 ± 4.59	34.15 ± 5.18	< 0.001	
Behavior				
Control	24.68 ± 2.14	23.84 ± 2.39	0.022	0.171
Intervention	24.12 ± 2.70	24.13 ± 2.31	0.998	

* Within-group comparison between pre- and post-training in each group

** Comparison of changes over time between the two groups

## Discussion

The main innovation of this research was the integration of the Health Belief Model (HBM) into a web-based educational program—an approach not previously reported in similar domestic studies.

The results showed that the HBM-based web intervention effectively improved participants’ knowledge and health beliefs regarding oral hygiene. Significant increases in perceived susceptibility, perceived severity, perceived benefits, and self-efficacy indicated the program’s positive influence on participants’ perceptions and motivation. These findings suggest that the structured, interactive nature of the web-based program helped participants link oral health information to their personal experiences, which may have enhanced internal motivation and cognitive engagement—core elements of the HBM framework [18, 19].

To the best of our knowledge, no prior study in Iran has implemented a web-based, HBM-guided intervention to promote oral health among pregnant women, highlighting the novelty of this research. Similar to our results, studies conducted in other countries [19–21] have reported that web-based educational programs can improve knowledge and health beliefs. However, in some studies, such as Bakhtiar et al. [22], improvements were also seen in the control group, possibly due to the Hawthorne effect or increased awareness from study participation. Taken together, the evidence suggests that remote educational interventions can effectively raise pregnant women’s awareness of oral health.

The significant rise in perceived susceptibility observed in this study suggests that participants gained a deeper understanding of the risks associated with poor oral hygiene. This aligns with the findings of Izadirad et al. [21] and Gholizadeh et al. [23], but differs from Ghaffari et al. [20], where the change was not significant. These differences may be due to variations in educational content, delivery methods, follow-up duration, or participant engagement.

The increase in perceived severity after the intervention also reflects a stronger awareness of the consequences of neglecting oral health. This indicates that using visual and real-life examples can make the perceived threat more concrete, thereby facilitating cognitive appraisal and emotional engagement, both of which are essential precursors for behavioral intention.

Enhancements in perceived benefits demonstrate the program’s success in emphasizing the value of preventive oral health behaviors. This finding is in line with prior studies, such as Ghaffari et al. [20], which stressed the importance of clearly presenting the tangible benefits of health-promoting actions.

Interestingly, perceived barriers also increased significantly in the intervention group. This pattern is common in educational interventions, as greater awareness may initially heighten recognition of challenges before individuals develop coping strategies. It highlights the importance of integrating problem-solving components into future programs to transform awareness of barriers into self-regulatory action. Similar findings have been reported in other studies [19, 23], suggesting that future interventions should include targeted components to address perceived barriers.

Although cues to action improved in the intervention group, the difference compared to the control group was not statistically significant. This may reflect the limited ability of web-based programs to provide sufficient behavioral prompts. Previous research has shown mixed results regarding this construct [24, 25], which may depend on the level of interactivity and engagement. Incorporating interactive reminders or personalized feedback might strengthen this effect.

The significant improvement in self-efficacy observed in this study supports the HBM framework, which identifies self-efficacy as a strong predictor of health behavior [26, 27]. Structured online education allows participants to practice skills, receive feedback, and build confidence. Similar results have been observed in other HBM-based interventions [19–23].

Although preventive behavior scores increased in the intervention group, the difference between groups was not statistically significant. This finding reinforces the notion that cognitive and belief changes precede behavioral modifications. According to the HBM, actual behavioral adoption often requires reinforcement and environmental cues beyond the initial educational exposure. This finding agrees with Bashirian et al. [24], who reported that health education programs do not always lead to behavioral change. Cognitive and attitudinal improvements are important but may not be sufficient to sustain long-term behavior without continued reinforcement, environmental support, and motivation.

Overall, our findings suggest that HBM-guided, web-based education is an effective and cost-efficient strategy for promoting oral health during pregnancy, particularly when face-to-face sessions are limited by time or location. Nevertheless, this study had several limitations. First, the data were self-reported, which may have introduced recall or social desirability bias, potentially leading to an overestimation of positive behaviors. Second, selection bias is a considerable limitation, as participation required internet access and basic digital literacy. This likely excluded individuals with lower socioeconomic status, thereby limiting the generalizability of our findings to the broader population of pregnant women. Third, the relatively short follow-up period (three months) restricted our ability to evaluate the long-term sustainability of the intervention’s effects on knowledge, beliefs, and behavior. Future studies should address these limitations by recruiting participants with varying levels of digital access, employing objective behavioral or clinical assessments, and implementing longer follow-up durations to better assess the long-term maintenance of behavioral changes.

## Data Availability

The datasets generated and analyzed during the current study are not publicly available due to privacy and ethical restrictions imposed by the Ethics Committee of Bushehr University of Medical Sciences, in order to protect the confidentiality of the pregnant women participants. However, de-identified data may be made available from the corresponding author upon reasonable request and with approval from the Ethics Committee.

## Abbreviations

HBM: Health Belief Model
WHO: World Health Organization
FGF: Fibroblast growth factor
BDNF: Brain-derived neurotrophic factor
CRP: C-reactive protein
PGE2: Prostaglandin E2
ICT: Communication Technology
HCP: Healthcare Professionals
SPSS: Statistical Package for the Social Sciences
